# Contrast media enhancement reduction predicts tumor response to presurgical molecular-targeting therapy in patients with advanced renal cell carcinoma

**DOI:** 10.18632/oncotarget.17930

**Published:** 2017-05-17

**Authors:** Shogo Hosogoe, Shingo Hatakeyama, Ayumu Kusaka, Itsuto Hamano, Yoshimi Tanaka, Kazuhisa Hagiwara, Hideaki Hirai, Satoko Morohashi, Hiroshi Kijima, Hayato Yamamoto, Yuki Tobisawa, Tohru Yoneyama, Takahiro Yoneyama, Yasuhiro Hashimoto, Takuya Koie, Chikara Ohyama

**Affiliations:** ^1^ Department of Urology, Hirosaki University Graduate School of Medicine, Hirosaki, Japan; ^2^ Department of Pathology and Bioscience, Hirosaki University Graduate School of Medicine, Hirosaki, Japan; ^3^ Department of Advanced Transplant and Regenerative Medicine, Hirosaki University Graduate School of Medicine, Hirosaki, Japan

**Keywords:** renal cell carcinoma, presurgical therapy, axitinib, radiological response

## Abstract

**Background and Objective:**

A quantitative tumor response evaluation to molecular-targeting agents in advanced renal cell carcinoma (RCC) is debatable. We aimed to evaluate the relationship between radiologic tumor response and pathological response in patients with advanced RCC who underwent presurgical therapy.

**Results:**

Of 34 patients, 31 underwent scheduled radical nephrectomy. Presurgical therapy agents included axitinib (*n* = 26), everolimus (*n* = 3), sunitinib (*n* = 1), and axitinib followed by temsirolimus (*n* = 1). The major presurgical treatment-related adverse event was grade 2 or 3 hypertension (44%). The median radiologic tumor response by RECIST, Choi, and CMER were −19%, −24%, and −49%, respectively. Among the radiologic tumor response tests, CMER showed a higher association with tumor necrosis in surgical specimens than others. Ki67/MIB1 status was significantly decreased in surgical specimens than in biopsy specimens. The magnitude of the slope of the regression line associated with the tumor necrosis percentage was greater in CMER than in Choi and RECIST.

**Materials and Methods:**

Between March 2012 and December 2016, we prospectively enrolled 34 locally advanced and/or metastatic RCC who underwent presurgical molecular-targeting therapy followed by radical nephrectomy. Primary endpoint was comparison of radiologic tumor response among Response Evaluation Criteria in Solid Tumors (RECIST), Choi, and contrast media enhancement reduction (CMER). Secondary endpoint included pathological downstaging, treatment related adverse events, postoperative complications, Ki67/MIB1 status, and tumor necrosis.

**Conclusions:**

CMER may predict tumor response after presurgical molecular-targeting therapy. Larger prospective studies are needed to develop an optimal tumor response evaluation for molecular-targeting therapy.

## INTRODUCTION

Targeted therapy agents used over the last decade for the treatment of metastatic renal cell carcinoma (RCC) have demonstrated significant improvements in survival; these agents include tyrosine kinase inhibitors (TKIs), mammalian target of rapamycin inhibitors (mTORis), and vascular endothelial growth factor receptor antibodies (bevacizumab) [1, 2]. While changes in tumor length (Response Evaluation Criteria in Solid Tumors: RECIST) is the standard for assessing tumor response, it does not capture central necrosis and devascularization effects of molecular-targeting agents. The Choi criteria [3] is one quantitative method that can be used to measure tumor response, but it is influenced by capturing the timing after the radiographic contrast media injection. To overcome this, we developed a method using contrast media enhancement reduction (CMER) that potentially captures viable tumors with the exclusion of central necrosis using computed tomography (CT). Because CMER measures the enhanced area alone, it has the potential to overcome time phase appearance differences before and after therapy.

Besides a radiologic response evaluation, a quantitative comparison between the radiologic response and pathologic necrosis poses the next challenge. There is an unmet need for a quantitative tumor response biomarker that corresponds with pathological outcomes. A presurgical setting is ideal to make comparisons between radiologic and pathologic responses. Although the role of presurgical molecular-targeting therapy for advanced RCCs has not been clearly established, several studies have suggested a survival benefit [4–11] that corresponds to accumulating evidences of efficacy and safety. Here, we investigated the clinical implication of CMER in patients with advanced RCC who underwent presurgical molecular-targeting therapy followed by radical nephrectomy, and compared radiologic tumor responses by RECIST, Choi, and CMER with pathological outcomes in those patients.

## RESULTS

### Patient characteristics and molecular-targeting agents

Of 34 patients, three patients refused surgery and withdrew from this clinical trial during the presurgical periods. Of 31 patients who underwent radical nephrectomy, presurgical therapy agents included sunitinib (*n* = 1), axitinib (*n* = 26), everolimus (*n* = 3), and axitinib followed by temsirolimus due to grade 2 heart failure (*n* = 1). The mean age was 67 ± 11 years old. Patients with inferior vena cava (IVC) thrombus and metastatic disease were 10 (32%) and 11 (35%), respectively. The median duration of presurgical therapy was 3.7 months (Table 1). Of 11 patients with metastasis, the number of patients with MSKCC favorable-risk, intermediate-risk, and poor-risk were 1, 9, and 1, respectively. Because one patients with poor-risk had a good general status with younger age (57 years) and oligometastatic small lesion in lung, we included in the present study. Mean relative dose intensity for sunitinib (*n* = 1), mTORi (*n* = 4), and axitinib (*n* = 26) were 100%, 100% and 95%, respectively. In the presurgical axitinib therapy, 5 and 2 patients experienced dose reduction (mean 30%) and escalation (mean 114%), respectively.

**Table 1:** Background of patients

Baseline patient characteristics	Presurgical
*n*	31
Age, years	67 ± 11
Sex (male), *n* =	20 (65%)
ECOG PS > 1, *n* =	3 (10%)
Cardiovascular disease, *n* =	5 (16%)
Diabetes Mellitus, *n* =	4 (13%)
Clinical T stage, *n* =	2.8 ± 0.8
Clinical T stage 3 or 4, *n* =	26 (84%)
IVC thrombus, *n* =	10 (32%)
Metastatic disease, *n* =	11 (35%)
Extent of metastases, *n* =	
Low volume	6 (55%)
High volume	5 (45%)
Perioperative outcomes	
Duration of presurgical therapy (months)	3.7 (3.1–4.6)
Duration of radical nephrectomy (min)	300 (154–515)
Blood loss (g)	147 (124–197)
Pathological T stage	2.4 ± 0.9
Pathological T stage 3 or 4, *n* =	20 (65%)
Clear cell subtype, *n* =	27 (87%)

### Comparison of radiologic responses

**Figure 1:**
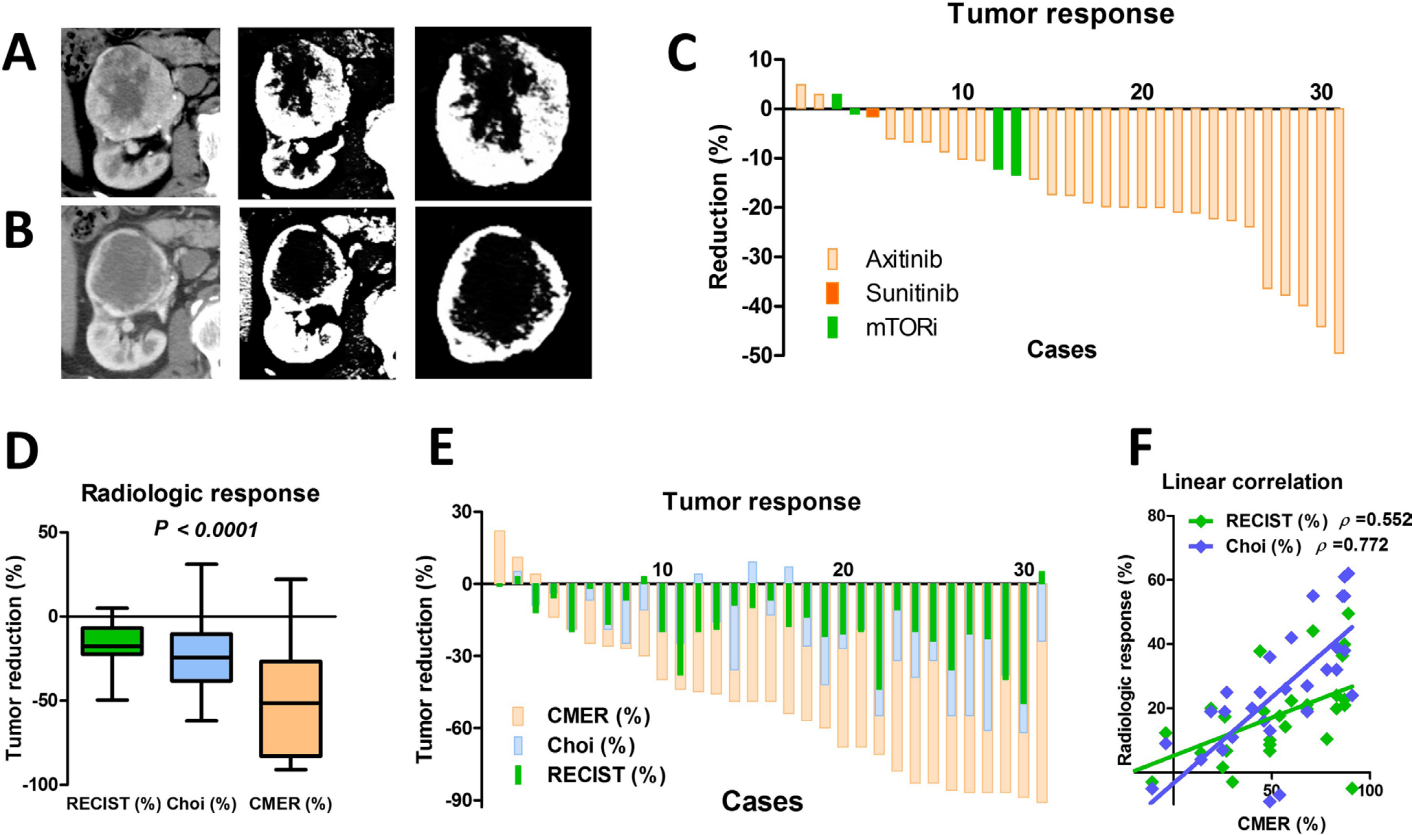
Radiological response evaluation Three tests were used to evaluate intratumor necrosis: RECIST, Choi, and contrast media enhancement reduction (CMER). The representative tumor responses before (**A**) and after (**B**) axitinib are shown. The tumor response was −12% and −74% for RECIST and CMER in this case, respectively. The waterfall plot of RECIST shows that the median response was −19% [interquartile range (IQR): −7% to −22%), and no patients experienced disease progression during the presurgical period (**C**). The radiological tumor reduction indicated by CMER was significantly higher than that indicated by RECIST (*P* < 0.001) or Choi (*P* < 0.001), although no statistical difference was observed between RECIST and Choi (*P* = 0.116) (**D**). Waterfall plots in three radiological tumor responses are shown (**E**). Linear regression analyses demonstrated the correlation among RECIST, Choi, and CMER values (**F**). The magnitude of the slope of the regression line to CMER was greater with Choi (0.535, Spearman *ρ* = 0.772) than with RECIST (0.238, Spearman *ρ* = 0.552).

Three tests were used to evaluate tumor responses: RECIST, Choi, and CMER. Figure 1 shows a case of tumor response before (Figure 1A) and after (Figure 1B) axitinib. A supplemental figure (Supplementary Figure 1) shows representative tumor responses. The waterfall plot of RECIST shows that the median response was −19% [interquartile range (IQR): −7% to −22%), and no patients experienced progressive disease (by RECIST) during the presurgical period (Figure 1C). The median tumor reduction in Choi and CMER were −24% (IQR: −9% to −38%) and −49% (IQR: −27% to −83%), respectively (Figure 1D). Although no significant difference was observed between RECIST and Choi (*P* = 0.116), tumor reduction was significantly higher with CMER than with RECIST (*P* < 0.001) or Choi (*P* < 0.001). The waterfall plots of RECIST, Choi, and CMER are shown on Figure 1E. The correlations among three radiological tumor responses were investigated by linear regression analyses (Figure 1F). The magnitude of the slope of the regression line to CMER was greater with Choi (0.535, Spearman *ρ* = 0.772) than with RECIST (0.238, Spearman *ρ* = 0.552).

### Correlation between radiologic response and pathological tumor necrosis

**Figure 2:**
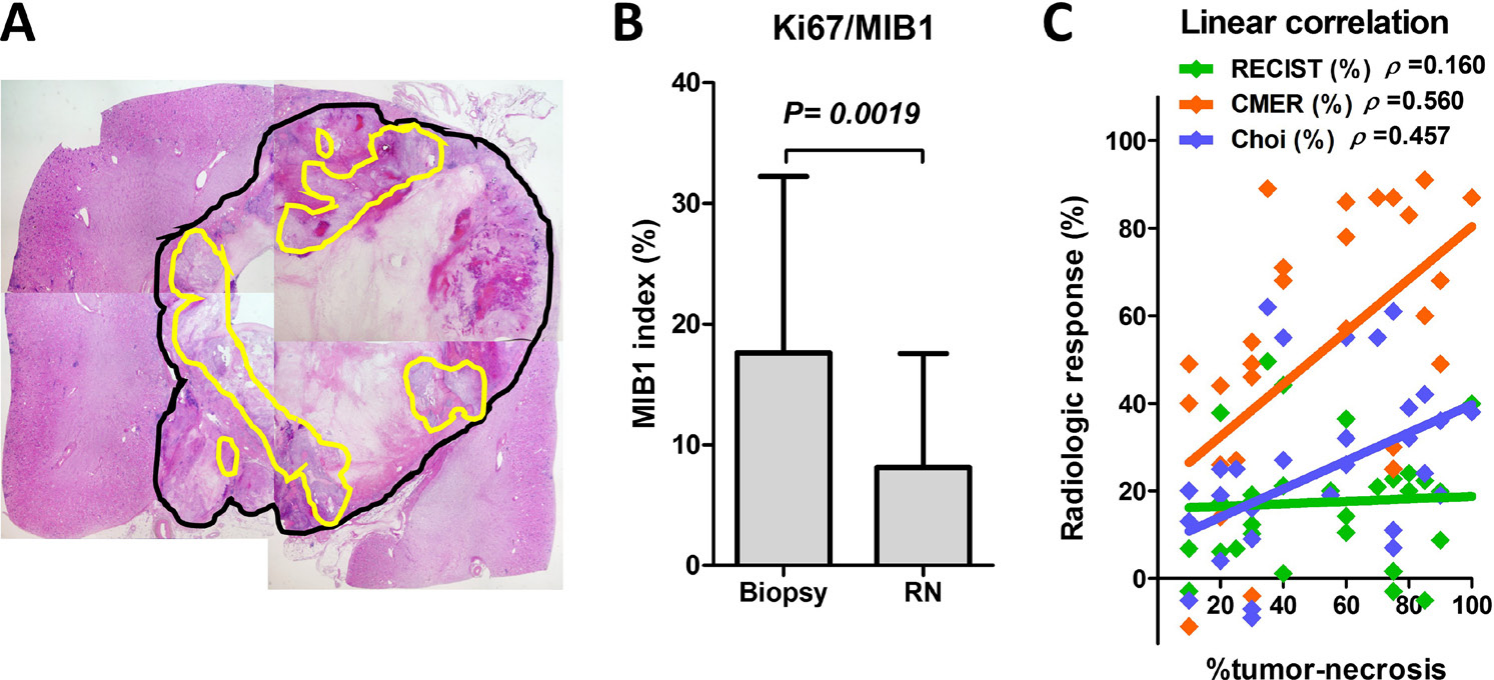
Pathological response evaluation from surgical specimens Representative pathological findings of radical nephrectomy specimens are shown (**A**). Residual viable cells in radical nephrectomy specimens were evaluated, and the ratios of the non-viable tumor areas were calculated as a percent of tumor necrosis (black line, all tumor; yellow line, viable cells). The Ki67/MIB1 index was significantly decreased in radical nephrectomy specimens versus needle biopsy specimens in the presurgical group (**B**). Linear regression analyses show the correlation between the percent of tumor necrosis and radiological response tests (**C**). The magnitude of the slope of the regression line associated with the percent of tumor necrosis was greater with CMER (0.599, Spearman *ρ* = 0.560, *P* = 0.003) than with Choi (0.321, Spearman *ρ* = 0.457, *P* = 0.025) and RECIST (0.321, Spearman *ρ* = 0.160, *P* = 0.025) (Spearman's correlation coefficient test).

Representative pathological findings of radical nephrectomy specimens are shown on Figure 2A where the black and yellow lines denote the tumor area and viable cell area, respectively. The Ki67/MIB1 index was significantly decreased in radical nephrectomy specimens (8.1%) versus needle biopsy specimens (18%) in the presurgical group (Figure 2B). Linear regression analyses showed correlations between the tumor necrosis percentage and radiological response tests (Figure 2C). The magnitude of the slope of the regression line associated with the percent of tumor necrosis was greater with CMER (0.599, Spearman *ρ* = 0.560, *P* = 0.003) than with Choi (0.321, Spearman *ρ* = 0.457, *P* = 0.025) and RECIST (0.321, Spearman *ρ* = 0.160, *P* = 0.025). One patient with an IVC thrombus (level 1) experienced a stage pT0 tumor after presurgical axitinib. The radiologic responses in RECIST, Choi, and CMER in this patient were −40%, −38%, and −87%, respectively.

### Adverse events

The majority of adverse events related to presurgical therapy were grade 1 or 2 (Supplementary Table 1). Grade 2 or 3 hypertension was the major adverse event during presurgical TKI administration (44%) followed by mild and temporally proteinuria (grade 1 or 2). One presurgical axitinib patient experienced cholecystitis (grade 3) induced by cholelithiasis 3 days before the planned surgery; radical nephrectomy and cholecystectomy were performed simultaneously. One patient with chronic kidney disease experienced lung edema (grade 3) after receiving presurgical axitinib. One patient experienced hyperglycemia (grade 3) after receiving presurgical everolimus. There were no grade 4 or 5 perioperative complications in the presurgical group. Grade 1 or 2 ileus was the major postoperative complication after presurgical therapy (Supplementary Table 2).

## DISCUSSION

A quantitative comparison between a radiologic response for molecular-targeting agents and pathologic necrosis is debatable. This is the first study to compare the antitumor effect between a radiologic response and pathological tumor response in RCC. The essential finding of the present study is that contrast media enhancement reduction might be linked with tumor necrosis and may be a useful tool for evaluating tumor shrinkage. Although the median radiological response using RECIST showed only a −19% reduction, a significantly higher CMER (−49%) and decrease in the Ki67/MIB1 index were observed in the presurgical group. Notably, one patient with an IVC thrombus (level 1) experienced a stage pT0 tumor after presurgical axitinib. Our finding is supported by the recent study [12]. Smith et al. suggested a similar concept in that an initial change in the vascular tumor burden, a measure of the vascularized tumor area by CT imaging, predicted tumor response to molecular-targeting therapy in patients with metastatic RCC treated with sunitinib [12]. These results suggest that a quantitative measurement of tumor vasculature has potential to predict not only the tumor response but also the prognosis. In the present study, we could not investigate the CMER impact on prognosis due to a small sample size and shorter follow-up periods. Because there is a lack of consensus regarding the standard protocol for shrinkage evaluation in presurgical therapy, further study is necessary to address the clinical implications of a quantitative measurement of tumor vasculature on prognosis.

Optimal timing, patient selection, and presurgical therapy regimen for molecular-targeting agents in patients with advanced RCCs also need to be debated. Corresponding to accumulating evidences for efficacy and safety, an interest in presurgical therapy using molecular-targeting agents for non-metastatic diseases [10], preservation of renal parenchyma associated with a partial nephrectomy [11], a venous tumor thrombus extending to the IVC [5, 6], and large unresectable tumors or metastatic diseases [4, 7, 8] are increasingly reported. However, no level 1 evidence has identified the role of presurgical therapy for advanced RCC and any benefit remains unclear. In addition, the use of presurgical targeted therapy to downsize RCC is not recommended in European Association of Urology guidelines [13]. Therefore, another prospective study is necessary to address the clinical benefit of presurgical therapy for advanced RCC.

The majority of patients in this study (84%) received presurgical axitinib. Our strategy was supported by several studies for the efficacy and safety of axitinib as first-line therapy [14, 15] and initial report of a Phase 2 prospective neoadjuvant axitinib trial [10]. They reported that axitinib was more favorable than sunitinib as a neoadjuvant therapy. Indeed, no patients in our study experienced disease progression, and the mean relative dose intensity of axitinib was 95%. In addition, the tumor response with axitinib may be more favorable in a presurgical setting compared with mTORi. The maximum radiological responses with axitinib and mTORi were determined to be −49.6% and −13.4%, respectively, using RECIST, and −91% and +4%, respectively, using CMER.

This study had several limitations. The sample size and study design prevented us from coming to a definitive conclusion. In addition, due to careful patient selection for presurgical therapy, we were unable to control for selection bias and other unmeasurable confounding factors. The feasibility of tumor necrosis as a surrogate marker for a radiologic response needs further investigation. Furthermore, due to the sample size and shorter follow-up, we could not address the impact of CMER on prognosis. Our next study should compare the oncological outcome between RECIST, Choi, and CMER. The small number of adverse events (especially in all grade diarrhea) in the present study comparing to the previously reported data of axitinib (approximately 50%) [15–17] was a limitation of the present study. Despite these limitations, our results support the potential benefit of presurgical therapy for selected patients.

In conclusion, a quantitative evaluation of a radiological response for molecular-targeting therapy using CMER provided accurate tumor responses that corresponded with tumor necrosis. Further, large-scale studies are necessary to identify the indications, clinical benefits, and standard protocol for presurgical therapy.

## MATERIALS AND METHODS

### Design and ethics statement

This prospective, single-center study on the use of molecular-targeting agents prior to radical nephrectomy in patients with locally advanced and/or metastatic RCCs was conducted in accordance with the ethical standards of the Declaration of Helsinki. It was approved by the Ethics Committee of the Hirosaki University Graduate School of Medicine (authorization number 2012-099) and was registered as clinical trial UMIN000025209.

### Patient selection

Between March 2012 and December 2016, we prospectively enrolled 34 RCC patients who underwent presurgical molecular-targeting therapy. Inclusion criteria were 1) 20 years or older, 2) in patients with advanced renal cell carcinoma without prior targeting therapy, 3) clinically T3-4, N1, or oligometastatic disease (not multiple, small, or resectable) who underwent tumor biopsy prior to targeting therapy, 4) agree to use molecular-targeting therapy prior to radical nephrectomy. Exclusion criteria were 1) untreated brain metastasis, 2) active gastrointestinal hemorrhage, 3) poor general health (ECOG PS > 2), 4) major concomitant disease, 5) other medical condition likely to result in death within 6 months after the treatment initiation, 6) clinical evidence of any of unstable diseases including cardiovascular, infectious, immune, nervous system disease, and other active malignancies that influenced on therapy for renal cell carcinoma, 7) history of drug, alcohol, or substance abuse, 8) any condition, limitation, or disease that could, in the judgment of the investigator, preclude evaluation of response to targeting therapy.

### Endpoints

Primary endpoint was comparison of radiologic tumor response among Response Evaluation Criteria in Solid Tumors (RECIST), Choi, and contrast media enhancement reduction (CMER). Secondary endpoint included pathological downstaging, treatment related adverse events, postoperative complications, Ki67/MIB1 status, and tumor necrosis.

### Evaluation of variables

The variables analyzed were age, gender, Eastern Cooperative Oncology Group performance status (ECOG PS), history of cardiovascular disease, diabetes mellitus, presurgical therapy agents, clinical T and N stage, thrombus level, and extent of metastases [low volume (defined as the presence of < 5 lung metastases within 2 cm, single bone lesions, or involvement of a single lymph node) or high volume]. Toxicity was prospectively recorded based on the National Cancer Institute Common Terminology Criteria for Adverse Events, version 4.0. All the patients underwent an open radical nephrectomy. Molecular-targeting agents were discontinued 48–72 h before radical nephrectomy. Surgical duration (min), blood loss, and postoperative complications (Clavien-Dindo classification) were reviewed.

### Radiographic assessment in the presurgical group

CT of the chest, abdomen, and pelvis was performed before and after presurgical therapy during the late arterial phase (35–40 s post-contrast media injection). CT features of the tumors were reviewed three times: before therapy, three months after the molecular-targeting agents, and just before surgery. Tumor response was analyzed using RECIST ver. 1.1 [18], Choi criteria [3, 7], and CMER. CMER was evaluated using The Digital Imaging and Communications in Medicine (DICOM) files, OsiriX ver. 5.0.2 (Newton Graphics, Inc., Sapporo, Japan), and Photoshop CC2017 (Adobe systems, Inc. CA, USA). DICOM files were read using OsiriX and changed to the window level and width of 53.00 and 108, respectively. Next, representable slices before and after molecular-targeting therapy were saved as image files. The reduction of the contrast media-enhanced tumor area was measured using Photoshop. Image files were read using Photoshop; the tumor was removed from the surrounding area and the enhanced tumor area (pixels) was measured. Representative CMER tumor responses are shown in Supplementary Figure 1.

### Immunohistochemistry of Ki67/MIB1 in the presurgical group

To evaluate the prognostic relevance of the Ki67/MIB1 proliferation marker, immunohistochemistry for Ki67/MIB1 was performed using 3 μm slices from paraffin-embedded specimens and a Histofine immunostaining kit (Nichirei Co. Ltd., Tokyo, Japan). The monoclonal antibody against Ki67 (MIB1) (Dako, Denmark) was used at the optimal dilution of 1:50. In all cases, 5–10 high-power fields (400-fold) were selected and at least 1000 cells were independently evaluated by two of the authors (SH and YH). The number of Ki67/MIB1-positive cells per 100 adrenocortical cells was designated as the labeling index (MIB1 index). The MIB1 index was compared between the pretreatment biopsy and radical nephrectomy specimens in the presurgical group.

### Evaluation of pathological tumor necrosis in radical nephrectomy specimens

Residual viable cells in the radical nephrectomy specimens was evaluated by two pathologists (HH and SM); the ratio of non-viable areas in all tumors was calculated as the percent of tumor necrosis. The relationship between tumor necrosis and radiological responses (RECIST, Choi, and CMER) was evaluated using linear regression analyses in the presurgical group.

### Statistical analysis

Statistical analyses of the clinical data were performed using SPSS ver. 24.0 (SPSS, Armonk, NY: IBM Corp.), GraphPad Prism ver. 5.03 (GraphPad Software, San Diego, CA, USA), and R ver. 3.3.2 (R Foundation for Statistical Computing, Vienna, Austria). Categorical variables were compared using Fisher's exact or a χ^2^ test. Quantitative variables were expressed as means and standard deviation or medians with an interquartile range (IQR). The differences between the groups were compared using a Student's *t*-test for normal distributions or Mann-Whitney *U*-test for non-normal distributions. A linear regression analysis was performed to evaluate the relationship among radiological tests and pathological tumor necrosis. The correlation was analyzed using Spearman's correlation coefficient. *P* values < 0.05 were considered statistically significant.

## SUPPLEMENTARY MATERIALS FIGURE AND TABLES



## Abbreviations

CMER: contrast media enhancement reduction
CT: computed tomography
IVC: inferior vena cava
IQR: an interquartile range
RCC: renal cell carcinoma
RECIST: Response Evaluation Criteria in Solid Tumors
TKI: tyrosine kinase inhibitors
DICOM: The Digital Imaging and Communications in Medicine
