# Supplementary material for: Contrast media enhancement reduction predicts tumor response to presurgical molecular-targeting therapy in patients with advanced renal cell carcinoma
Source: Oncotarget. 2017 May 17;8(30):49749–56. doi: 10.18632/oncotarget.17930 (PMC5564804; doi:10.18632/oncotarget.17930)
Supplement: Supplementary file 1 [file oncotarget-08-49749-s001.pdf]

Contrast media enhancement reduction predicts tumor response to presurgical molecular-targeting therapy in patients with advanced renal cell carcinoma

SUPPLEMENTARY MATERIALS

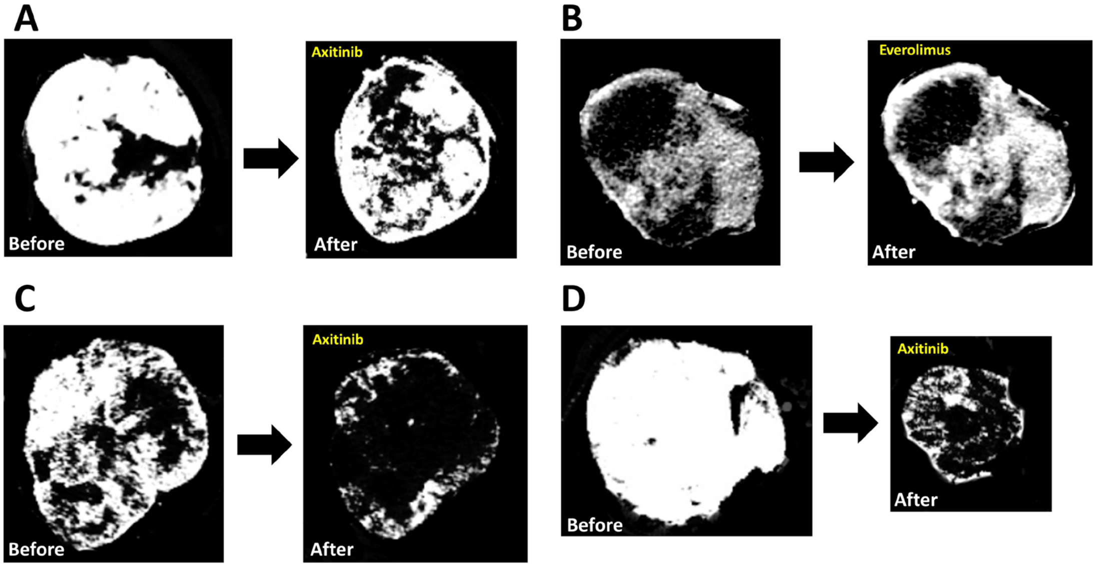

Supplementary Figure 1: Representative tumor response evaluated by CMER. Radiological responses between RECIST and CMER are shown. The case of presurgical axitinib showed a 7% and 27% tumor reduction by RECIST and CMER, respectively (**A**). The case of presurgical everolimus showed a 1.1% reduction by RECIST but 22% tumor progression was observed by CMER (**B**). The case of presurgical axitinib showed an 11% and 78% tumor reduction by RECIST and CMER, respectively (**C**). The case of presurgical axitinib showed a 39% and 87% tumor reduction by RECIST and CMER, respectively (**D**). This patient experienced a pT0 tumor 4.6 months after presurgical axitinib.

Supplementary Table 1: Adverse events related to presurgical molecular-targeting therapy

**TKI (*n* = 27)**	**All, *n* =**	**G1 or 2, *n* =**	**G3, *n* =**
Hypertension	12 (44%)	7	5
Proteinuria	6 (22%)	6	
Hypothyroidism	4 (15%)	4	
Fatigue	3 (11%)	3	
Hand-foot syndrome	3 (11%)	3	
Acute kidney injury	3 (11%)	2	1
Appetite loss	2 (7.4%)	2	
Abdominal pain	1 (3.7%)	1	
Diarrhea	1 (3.7%)		1
Heart failure	1 (3.7%)	1	
Lung edema	1 (3.7%)		1
Cholecystitis	1 (3.7%)		1
Liver dysfunction	1 (3.7%)	1	
Anemia	1 (3.7%)	1	
Hoarseness	1 (3.7%)	1	
mTORi (*n* = 4)			
Hyperglycemia	1 (25%)		1
Oral mucositis	1 (25%)	1	
Thrombocytopenia	1 (25%)	1	
Interstitial pneumonia	1 (25%)	1	

Supplementary Table 2: Post-operative complications (clavien-dindo classification)

	***n* =**	
Any grade	8 (26%)	
Grade 1 or 2	7 (23%)	
Grade 3	1 (3.2%)	
Grade 5	0 (0%)	
Description of complication	*n* =	Grade
Ileus	4 (13%)	1, 1, 1, 2
Acute kidney injury	1 (3.2%)	3
Deep vein thrombus	1 (3.2%)	2
Liver dysfunction	1 (3.2%)	2
Superficial wound dehiscence	1 (3.2%)	1
